# Wnt/β-Catenin Target Genes in Colon Cancer Metastasis: The Special Case of L1CAM

**DOI:** 10.3390/cancers12113444

**Published:** 2020-11-19

**Authors:** Sanith Cheriyamundath, Avri Ben-Ze’ev

**Affiliations:** Department of Molecular Cell Biology, Weizmann Institute of Science, Rehovot 76100, Israel; sanith.cheriyamundath@weizmann.ac.il

**Keywords:** L1, Wnt target genes, β-catenin, cell adhesion, colon cancer, NF-κB, invasion and metastasis, cancer stem cells, EMT, Lgr5

## Abstract

**Simple Summary:**

The Wnt/β-catenin cell–cell signaling pathway is one of the most basic and highly conserved pathways for intercellular communications regulating key steps during development, differentiation, and cancer. In colorectal cancer (CRC), in particular, aberrant activation of the Wnt/β-catenin pathway is believed to be responsible for perpetuating the disease from the very early stages of cancer development. A large number of downstream target genes of β-catenin-T-cell factor (TCF), including oncogenes, were detected as regulators of CRC development. In this review, we will summarize studies mainly on one such target gene, the L1CAM (L1) cell adhesion receptor, that is selectively induced in invasive and metastatic CRC cells and in regenerating cells of the intestine following injury. We will describe studies on the genes activated when the levels of L1 are increased in CRC cells and their effectiveness in propagating CRC development. These downstream targets of L1-signaling can serve in diagnosis and may provide additional targets for CRC therapy.

**Abstract:**

Cell adhesion to neighboring cells is a fundamental biological process in multicellular organisms that is required for tissue morphogenesis. A tight coordination between cell–cell adhesion, signaling, and gene expression is a characteristic feature of normal tissues. Changes, and often disruption of this coordination, are common during invasive and metastatic cancer development. The Wnt/β-catenin signaling pathway is an excellent model for studying the role of adhesion-mediated signaling in colorectal cancer (CRC) invasion and metastasis, because β-catenin has a dual role in the cell; it is a major adhesion linker of cadherin transmembrane receptors to the cytoskeleton and, in addition, it is also a key transducer of Wnt signaling to the nucleus, where it acts as a co-transcriptional activator of Wnt target genes. Hyperactivation of Wnt/β-catenin signaling is a common feature in the majority of CRC patients. We found that the neural cell adhesion receptor L1CAM (L1) is a target gene of β-catenin signaling and is induced in carcinoma cells of CRC patients, where it plays an important role in CRC metastasis. In this review, we will discuss studies on β-catenin target genes activated during CRC development (in particular, L1), the signaling pathways affected by L1, and the role of downstream target genes activated by L1 overexpression, especially those that are also part of the intestinal stem cell gene signature. As intestinal stem cells are highly regulated by Wnt signaling and are believed to also play major roles in CRC progression, unravelling the mechanisms underlying the regulation of these genes will shed light on both normal intestinal homeostasis and the development of invasive and metastatic CRC.

## 1. Introduction

Cell–cell adhesion is a basic biological process in multicellular organisms that determines cellular and tissue morphogenesis, and its disruption is a hallmark of cancer development. Aberrant signaling mediated by changes in cell–cell adhesion is a characteristic feature of invasive and metastatic cancer cells. Wnt/β-catenin signaling is a key signaling pathway that is hyperactivated in the majority of inherited colorectal cancer (CRC) patients and serves as a very useful model for studying adhesion-mediated mechanisms underlying CRC development [1,2]. This notion is supported by findings demonstrating that β-catenin plays a dual role in the cell. It is a major linker of cell–cell adhesion receptors (of the cadherin type) to the actin-cytoskeleton and, in addition, β-catenin plays a critical role in transmitting the Wnt signal to the nucleus by being a co-transcriptional activator [together with T-cell factor (TCF)] of Wnt target genes in the nucleus [3,4]. These two seemingly unrelated roles of β-catenin and the characteristic hyperactivation of Wnt/β-catenin signaling in CRC can serve as a useful system for investigating the roles of adhesion-mediated and Wnt signaling in CRC invasion and metastasis.

Wnt signaling was discovered over 40 years ago [5,6] and was first shown to play a role in determining the segmentation pattern in *Drosophila* [7]. Following these original studies, in the coming years, a role for Wnt signaling in embryonic axis determination in vertebrates was reported [8], and the potential involvement of the Wnt pathway in cancer development in humans was suggested [9]. In parallel, numerous studies addressed the identification of downstream components in the Wnt signaling pathway and discovered that inactivating mutations in the adenomatous polyposis coli (APC) gene, which is involved in β-catenin degradation, is a key step in the activation of Wnt signaling during CRC development [10]. In addition, stabilizing mutations in β-catenin against degradation by the ubiquitin-proteasomal system were also identified in a minority of CRC cases [11,12]. At this stage, an important avenue of research consisted of unraveling the target genes of Wnt/β-catenin signaling that are responsible for human CRC development. As the early steps in tumorigenesis are driven by changes that lead to uncontrolled proliferation of cells, initial studies focused on asking whether key regulators of the cell cycle (especially those leading to increased cell proliferation) are target genes of Wnt signaling and contain β-catenin/TCF binding sites in their promoter region. These studies led to the discovery of c-myc [13] and cyclin D1 [14,15] as target genes of β-catenin/TCF transactivation. Since then, hundreds of additional β-catenin-TCF target genes were discovered; for most of these genes, their role in CRC development remains to be determined [16]. Initial immunohistochemical studies of human CRC tissue did not detect a significant accumulation of β-catenin in the nuclei of early-stage CRC tissue and β-catenin localization remained mostly at cell–cell junctions in both normal colonic epithelial cells and in differentiated areas of CRC tissue [17,18]. However, at the later stages of CRC development, especially during the invasive and metastatic stages of tumor progression, a vast accumulation of β-catenin could be demonstrated, mostly in the nuclei of cancer cells [17,18], in addition to a specific expression of β-catenin target genes at the invasive areas of the tumor [19].

In this review, we will describe studies mainly on one such β-catenin-TCF target gene, the neuronal cell adhesion receptor L1CAM (L1) and its downstream targets, and its role in CRC invasion and metastasis. We will also discuss studies suggesting that some genes induced by L1 overexpression are known genes of the colonic stem cell signature that control the homeostasis of the intestinal stem cell compartment. Because CRC is believed to originate from tumorigenic intestinal stem cells [20], we hope that studies on L1 and downstream Wnt/β-catenin target genes will provide novel insights into the control of normal intestinal homeostasis and will also provide new targets for CRC therapy.

## 2. Members of the L1 Family of Cell Adhesion Receptors Are β-Catenin-TCF Target Genes

Initial DNA microarray analyses of genes induced by activated β-catenin-TCF signaling in cancer cells identified two members of the L1 family of immunoglobulin-like cell adhesion receptors, NrCAM [21,22] and L1 [19]. These findings were unexpected because both L1 and NrCAM were known to be present mostly in nerve cells, playing key roles during brain development by regulating a number of dynamic cellular processes including axonal growth, fasciculation, and pathfinding [23,24]. In previous studies, numerous point mutations were discovered in the L1 molecule that have severe consequences on brain development, leading to mental retardation by a group of syndromes known as L1 syndrome, MASA syndrome, and X-linked hydrocephalus [25,26,27,28,29].

**Figure 1:**
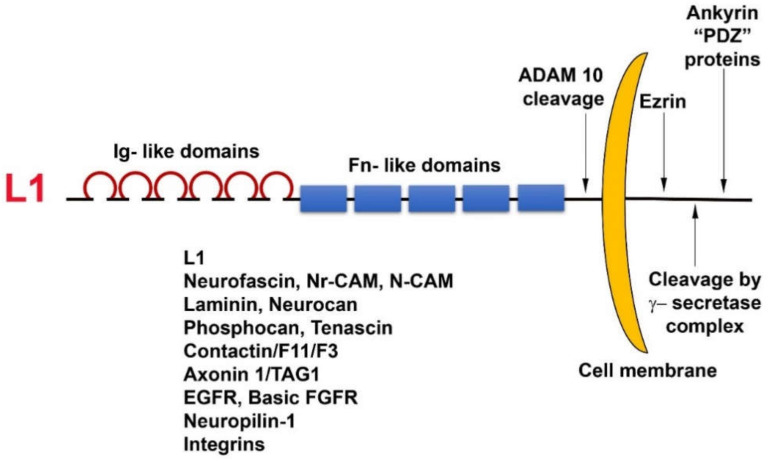
Domain structure and binding partners of L1. Note the numerous types of L1 ligands in the ectodomain as well as in the cytoplasmic tail domain of the molecule.

L1 is a cell adhesion transmembrane receptor, believed to act mostly by homophilic interactions with L1 on the surface of neighboring cells. L1 belongs to the superfamily of immunoglobulin-like cell adhesion receptors, containing six Ig-like domains and five fibronectin type III repeats; a transmembrane sequence; and a highly conserved (from *C. elegans* to man) cytoplasmic tail that has binding sites for ezrin, ankyrin, and other PDZ-containing proteins (Figure 1). In addition, L1 can be cleaved in the juxtamembrane region, outside the cell, by the metalloprotease ADAM10, and inside the cell, it has binding sites for the γ-secretase cleavage complex (Figure 1). Unlike cadherins that are characterized by strong homophilic interactions, L1 can interact via both homophilic and heterophilic binding to other neuronal cell adhesion molecules including neurocan, neuropilin1, axonin, and N-CAM [30]. In addition, L1 can associate with ECM components (fibronectin, laminin, tenascin) and ECM receptors (integrins) and can also bind to growth factor receptors, such as EGFR and basic FGFR [31]. Because of these numerous weak interactions of L1 with a variety of molecules, an increase in the expression of L1 in cancer cells could be advantageous for promoting the motile, invasive, and metastatic stages of tumorigenesis.

## 3. The Roles of L1 in Promoting CRC Cell Proliferation, Motility, Tumorigenesis, and Metastasis

Overexpression of L1 in 3T3 mouse fibroblasts and in human CRC cell lines results in elevated cell proliferation under stress (in the absence of serum); increased motility; invasion; tumorigenesis upon s.c injection into mice [19]; and, in the case of CRC cells, metastasis to the liver, a hallmark of human CRC progression [32]. The metalloprotease ADAM10, also a target gene of β-catenin-TCF transactivation, cleaves the ectodomain of L1 (Figure 1), thereby leading to its shedding, and promotes the rebinding of the shed L1 ectodomain to L1 molecules on the cell surface and enhances the metastatic potential of human CRC cells [32].

**Figure 2:**
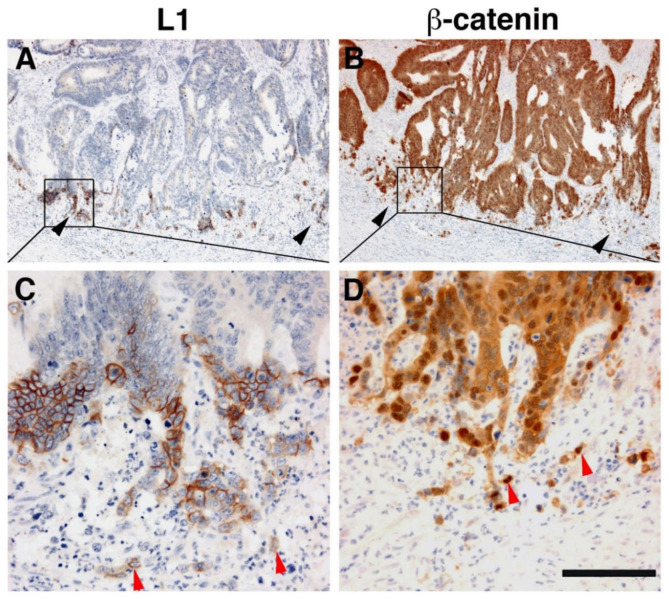
L1 is exclusively expressed at the invasive front of human colorectal cancer (CRC) tissue in cells expressing β-catenin in their nuclei. (**A**) Immunohistochemical staining of human CRC tissue for L1. Note the preferential localization of L1 in invasive areas of the tumor (black arrowheads), but not in the inner more differentiated areas of the tumor. (**B**) In contrast to L1 localization, a serial tissue section stained with anti β-catenin antibody displays a uniform staining of the same CRC tissue area. (**C**) Enlarged picture of the boxed area in (**A**) showing the membranal localization of L1. Single CRC cells invading into the stroma could also be seen (red arrowheads). (**D**) Magnified picture of the boxed area shown in (**B**) localizing β-catenin staining in both the cytoplasm and nuclei of CRC tissue cells and in the nuclei of single invasive cells (red arrowheads) at the tumor tissue edge [19]. Scale bar: (**A**,**B**) 375 μm, (**C**,**D**) 75 μm.

Immunohistochemical analysis of human CRC tissue revealed that the more differentiated areas of the tumor and the normal colonic epithelium do not express L1 [19]. L1 is exclusively expressed at the invading edge of human CRC tissue (Figure 2) in the membrane of cells that display strong nuclear β-catenin staining, indicative of a highly active β-catenin-TCF transactivation [19]. These results were recently confirmed and extended to show that, while L1 is not required for adenoma initiation, it plays multiple roles in cancer propagation, liver metastasis, and chemoresistance [33]. This study also demonstrated that L1 is not expressed in the homeostatic intestinal epithelium, but its expression is required for CRC organoid formation and metastasis initiation and growth. Finally, L1 expression was shown to be crucial for the regrowth occurring during wound healing in the intestine following injury [33]. Taken together, these results are reminiscent of the important roles played by L1 in the dynamic cellular processes occurring in nerve cells during brain development (i.e., axonal growth, pathfinding, and fasciculation) [34].

## 4. Mechanisms and Downstream Targets of L1 Signaling

**Figure 3:**
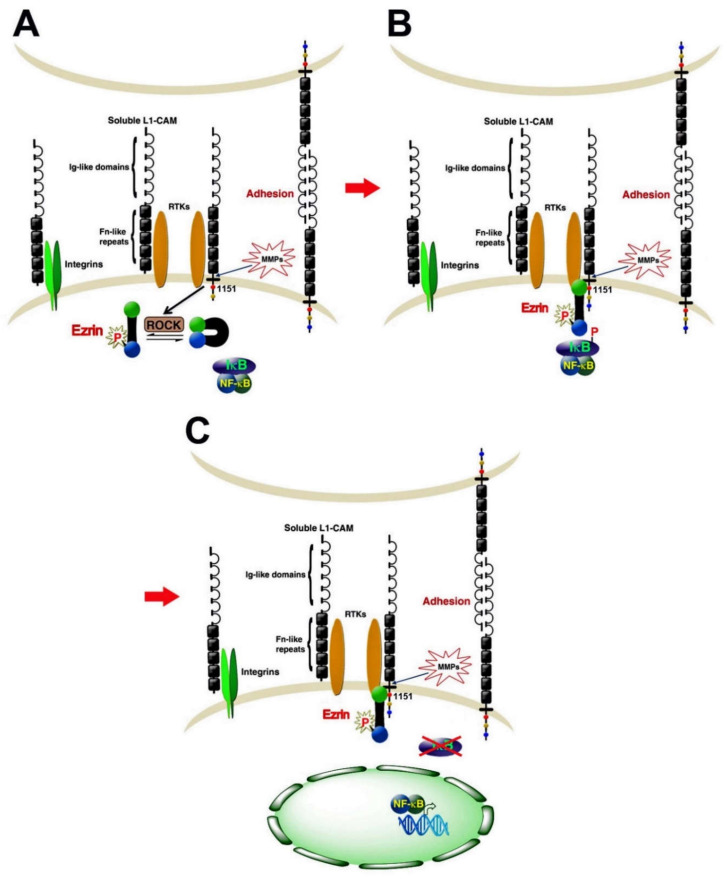
An NF-κB-ezrin signaling pathway is involved in L1 signaling in CRC cells. (**A**) The cytoskeletal protein ezrin is recruited to the cytoplasmic tail of L1 after it is activated by ROCK phosphorylation. The binding of activated ezrin to L1 involves Tyr1151 on the L1 cytoplasmic tail. (**B**) The L1-activated ezrin complex recruits the cytoplasmic NF-κB–IκB complex and leads to increased phosphorylation of IκB. (**C**) Elevated IκB phosphorylation results in its increased degradation by the proteasome and the release of NF-κB from the complex followed by NF-κB migration into the nucleus and transcriptional activation of target genes [40].

Numerous studies have addressed the mechanisms underlying the downstream signaling of L1. In neuronal cells, neurite outgrowth was shown to involve the MAPK pathway by increasing the expression of MAP2 [35]. In addition, the involvement of PI3K, ERK, and Rac-1 was also implicated in L1 signaling [36,37,38,39]. More recent studies have shown that, in CRC cells [40] and in pancreatic cancer cells [41], the signaling downstream of L1 involves the NF-κB pathway. According to this model (Figure 3), the L1 signaling pathway includes the activation of the cytoskeletal protein ezrin by phosphorylation on Thr567 (by ROCK), which leads to the re-localization of ezrin from filopodia to L1 in the membrane domain (Figure 3) and requires Tyr1151 on the L1 cytodomain (Figure 3B). Point mutations in Tyr1151 abolish the tumorigenic and metastatic capacities conferred by L1 [40]. In the next step, the IκB–NFκB complex is recruited to this multimolecular assembly, which enhances IκB phosphorylation and its degradation by the proteasome, thereby releasing NF-κB from IκB, which enables the migration of NF-κB into the nucleus and the activation of NF-κB target genes (Figure 3C). In support of this model, the activated (phosphorylated) p65 NF-κB subunit was detected in the nuclei of CRC tissue cells in invasive areas of the tumor together with L1 and ezrin expression in the membrane and cytoplasm of the same cells [40]. In addition, blocking NF-κB signaling in CRC cells expressing elevated L1 expression abolishes the properties conferred by L1 including enhanced growth and motility, tumorigenesis, and metastasis [40].

## 5. Genes Induced or Suppressed by L1 That Affect CRC Progression

In the next step, we wished to determine the genes induced, or suppressed, by L1 in CRC cells via an NF-κB-dependent mechanism using cDNA microarrays and compared these gene expression patterns to those of a large set of human CRC tissue samples [42]. A rather unexpected result of these analyses was the finding that, among the genes whose expression was suppressed by L1 overexpression (and by NF-κB signaling), and was also suppressed in human CRC tissue samples, was the well-known oncogene c-KIT [42]. Reconstitution of c-KIT expression in human CRC cell lines overexpressing L1 resulted in the inhibition of the pro-metastatic properties promoted by L1 in these cells [42]. The mechanism underlying this anti-metastatic effect conferred by c-KIT also involves the NF-κB pathway, but in this case, NF-κB plays an inhibitory role by suppressing the expression of SP-1, a key transcription factor of the c-KIT gene. The inhibition of SP-1 expression resulted in decreased c-KIT levels. In addition, the reduction in c-KIT also promoted an elevation in E-cadherin levels, the growing of cells in flat epithelial-like colonies, and the inhibition of SLUG (a key transcription factor of the EMT process), suggesting a mesenchymal to epithelial conversion (MET) [42]. While these dramatic effects of c-KIT on metastasis and cell motility indicated a tumor suppressive effect played by c-KIT [42], the proliferation in vitro and in vivo (in mice) of c-KIT overexpressing CRC cells showed that c-KIT enhances tumorigenesis, thus pointing to distinct modes of action of c-KIT in early versus late phases of tumor progression. A similar result was also reported for the key oncogene c-myc [43], thus further arguing that separate pathways mediate the tumorigenic and metastatic processes by these oncogenic molecules.

**Figure 4:**
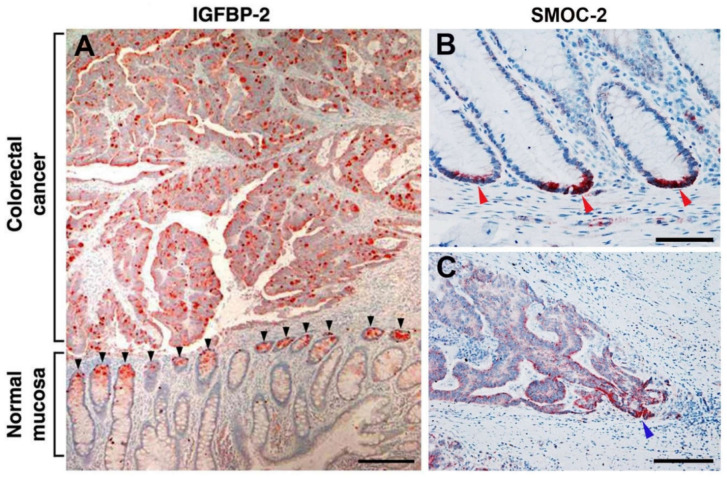
Genes induced by L1 in CRC cells include intestinal stem cell signature genes. (**A**) Insulin like growth factor receptor 2 (IGFBP-2) staining of CRC tissue revealed strong staining of the tumor tissue throughout the tissue, while in the adjacent normal mucosa, IGFBP-2 staining was exclusively confined to the bottom of colonic crypts (black arrowheads). (**B**) The intestinal stem cell signature gene secreted modular calcium-binding matricellular protein-2 (SMOC-2) was detected at the bottom of colonic crypts in normal colonic mucosa (red arrowheads). (**C**) In CRC tissue, SMOC-2 was localized in more differentiated areas of the tumor with stronger staining of invasive areas of the tumor (blue arrowhead) [44,46]. Scale bar: (**A**) 250 μm, (**B**) 50 μm, (**C**) 100 μm.

Further insight into the nature of genes induced by L1 in CRC cells by the NF-κB-ezrin pathway and their role in CRC tumorigenesis was provided by the discovery of insulin like growth factor receptor 2 (IGFBP-2) among these genes [44]. IGFBP-2 overexpression mimics the effects conferred by L1 on cell proliferation, motility tumorigenesis and metastasis, and the suppression of IGFBP-2 levels in L1-overexpressing cells blocked these properties conferred by L1 in CRC cells [44]. Interestingly, IGFBP-2 forms a molecular complex with L1, further supporting the important role played by these molecules in CRC progression. A most significant finding regarding the possible role of IGFBP-2 in CRC was derived from immunohistochemical analyses of CRC tissue samples to detect the localization of IGFBP-2. We detected IGFBP-2 at increased levels throughout the human CRC tissue samples, co-localizing with the activated p65 NF-κB subunit [44]. Most importantly, in the adjacent normal colonic mucosa, IGFBP-2 was exclusively localized at the bottom of the colonic crypts (Figure 4A). Because cells in the colonic crypts, especially at the crypts bottom, contain the colonic stem cells [45], which are believed to also be progenitors of the developing human CRC [45], we have further investigated this relationship between L1-induced genes and the colonic stem cell signature genes.

## 6. Colonic Stem Cell Signature Genes Induced by L1 in CRC Cells

The human intestinal epithelium contains invaginating crypts that harbor, at their base, the intestinal stem cells that express the Lgr5 molecular marker [20]. This epithelium is the most frequently regenerating tissue in the body and the lifetime of intestinal epithelial cells is less than a week. The stem cells fuel a continuous generation of all differentiated colonic cell types and are believed to be the progenitors of human CRC cells [47]. Among the genes induced by L1-ezrin-NF-κB signaling, we detected (in addition to IGFBP-2) the secreted modular calcium-binding matricellular protein-2 (SMOC-2). SMOC-2 is known as a representative of the group of Lgr5^+^ intestinal stem cell signature genes in mice [48]. We found that the induction of SMOC-2 in human CRC cells was necessary for the pro-tumorigenic properties conferred by L1 [46]. SMOC-2 overexpression could mimic the increase in cell proliferation under stress, motility, tumorigenesis, and liver metastasis and confers a more mesenchymal phenotype characterized by suppression of E-cadherin levels and an increase in the EMT-promoting transcription factor SNAIL. These properties of SMOC-2 overexpressing in CRC cells involve signaling by integrin linked kinase (ILK) [46]. In addition, we found an increase in the intestinal stem cell signature gene Lgr5 in CRC cells overexpressing SMOC-2, L1, or the p65 subunit of NF-κB [46]. Most significantly, we detected SMOC-2 exclusively at the base of normal colonic epithelial crypts (Figure 4B) and a preferential increase in its expression at invasive areas of human CRC tissue (Figure 4C).

In the next step, we identified clusterin (CLU) as a gene induced by L1 that is also expressed at increased levels in Lgr5^+^ intestinal stem cells of the mouse [49]. CLU is a secreted highly glycosylated protein that was implicated in playing a role in a variety of human tumors and is considered to be a marker for CRC development [50]. Similar to IGFBP-2 and SMOC-2, CLU overexpression induces CRC motility and tumorigenesis, but CLU does not promote experimental liver metastasis, implying the involvement of additional factors. However, the suppression of CLU in L1-overexpressing cells dramatically reduced their metastatic potential [49]. The mechanism of L1-mediated increase in CLU does not involve the NF-κB pathway, but rather a STAT-1-mediated elevation in the expression of the transcription factor SP-1 that activates the CLU gene promoter [49].

In the search for key intestinal/colonic stem cell compartment signature genes that could be activated by L1-mediated signaling in CRC, we turned to analyze the expression of ASCL2 in L1-overexpressing CRC cells. ASCL2 is a basic-helix-loop-helix transcription factor, a target gene of Wnt/β-catenin signaling and is restricted to Lgr5^+^ basal crypt cells in both mice and humans [48]. A recent study identified ASCL2 as the key transcriptional regulator that is induced at a very early stage during regeneration of the intestinal stem cell compartment following injury [51]. We found that overexpression of L1 in CRC cells induces the expression and nuclear accumulation of ASCL2, a decrease in E-cadherin levels, and increased levels of β-catenin in the nucleus, together with elevated β-catenin-TCF transactivation of Wnt/β-catenin target genes [52]. This downregulation of E-cadherin expression, the increase in the accumulation of nuclear β-catenin, and the transactivation of Wnt/β-catenin-TCF target genes were also reported in breast cancer cells [53]. This suggests that the replacement of E-cadherin-mediated adhesions by L1 in CRC cells is a more general characteristic of cancer cells. In addition, we found that the overexpression of ASCL2 in CRC cells could mimic the effects conferred by L1 on cell proliferation, motility, tumorigenesis, and liver metastasis (including an elevation in the intestinal stem cell signature genes Lgr5, OLFM4, and SMOC-2), while ASCL2 suppression in L1-transfected CRC cells blocked these properties conferred by L1 [52]. We detected ASCL2 in invasive areas of human CRC tissue in cells expressing increased levels of L1 (but not in normal colon mucosa) [52], indicating that L1 and ASCL2 cooperate in promoting CRC progression.

## 7. Genes Affected by Point Mutations in the L1 Ectodomain That Regulate CRC Development

As inherited mutations in the L1 ectodomain were shown to affect the adhesive properties of L1 and are associated with severe human brain developmental diseases [25,26,27,28,29,54], we searched for genes induced by L1 that are affected by specific point mutations in the L1 ectodomain and examined their role in CRC development. All the known ectodomain point mutants of L1 that we analyzed lost their ability to confer the tumorigenic and metastatic properties in CRC cells [55]. Among the genes that are specifically affected by the L1/H210Q mutation, but not by other L1 mutations in the L1 ectodomain, we identified the membrane-associated neutral endopeptidase, neprilysin (CD10) [55]. We found that the induction of CD10 by L1 that is blocked by the L1/H210Q mutation is required for the pro-tumorigenic and metastatic capacities conferred by L1 [55]. As with several other L1-induced genes, CD10 expression was dependent on an NF-κB-ezrin signaling pathway and we identified L1 and CD10 in cells localized in invasive areas of CRC tissue, suggesting that the two molecules act together in promoting the invasive properties of CRC cells [55]. The identification of genes that are specifically affected by such L1 ectodomain point mutations could provide additional targets for CRC diagnosis and therapy.

## 8. Secreted Factors That Promote the Tumorigenesis Induced by L1 Overexpressing CRC Cells 

Because a great number of genes that are induced by L1 overexpression in CRC cells are coding for either membrane bound proteins and are exposed to the extracellular milieu, or proteins secreted into the culture medium (see above, IGFBP-2, CLU, neprilysin, SMOC-2), we conducted a proteomic analysis of the secretome from L1 expressing CRC cells. Among the proteins whose levels were increased by L1 expression in CRC cells, we studied the role of the aspartate protease cathepsin D (CTSD), a lysosomal and secreted protein, because numerous studies reported on its association with the development of cancer in various tumors [56,57,58,59,60,61]. The levels of RNA, protein, and secreted CTSD protein were increased in response to L1 expression, and this induction of CTSD was necessary for L1-mediated CRC progression and liver metastasis [62]. The overexpression of CTSD in CRC cells, in the absence of L1, could confer increased proliferation, motility, tumorigenesis, and liver metastasis in these cells [62]. Enhancing Wnt/β-catenin signaling increased the levels of CTSD, suggesting its involvement in regulating CTSD expression. CTSD was detected in more invasive areas of the tumor in both epithelial cells and the adjacent stromal compartment, but not in normal mucosa, supporting a role for CTSD in L1-mediated CRC progression [62].

Another protein whose level is elevated in the secretome of CRC cells is the ubiquitin-like interferon induced gene 15 (ISG15), which operates much like ubiquitin by conjugating to target proteins (ISGylation) [63]. We found that increased ISG15 levels were required for L1-mediated CRC progression because suppression of ISG15 expression blocked the L1-mediated increase in CRC cell motility, tumorigenesis, and metastasis [63]. The induction of ISG15 was dependent on proper L1–L1 mediated adhesions, as point mutations in the L1 ectodomain abolished its ability to induce the expression of ISG15 [63]. The induction in ISG15 by L1 was dependent on the NF-κB pathway and ISG15 was detected in CRC tumor tissue and in the adjacent stroma, but not in normal colonic mucosa, suggesting that ISG15 could serve as a therapeutic target for CRC treatment [63].

## 9. Conclusions

L1, a cell adhesion receptor and a target gene of Wnt/β-catenin signaling, is a key perpetuator of CRC development and metastasis. L1 is not expressed in normal homeostatic colonic mucosa, but is induced at the invasive front of CRC tissue in cells expressing the Lgr5 intestinal stem cell marker as well as during regeneration of the intestinal/colonic tissue following injury. In addition, L1 was reported to contribute to the generation of an immunosuppressive tumor microenvironment [64] and promotes chemoresistance [33,65,66]. The studies summarized above point to the numerous genes that are induced (and suppressed) during CRC progression following L1 expression. Because the level of L1 expression was shown to be a powerful prognostic factor for indicating poor survival in a variety of cancer types, L1 is considered a promising target for cancer therapy that involves blocking L1 antibodies in combination with cytostatic drugs and/or radio-immunotherapy [67,68,69,70]. The downstream target genes of L1-mediated CRC progression described here could mimic the effects conferred by L1 on the motile, tumorigenic, and metastatic properties of CRC cells. Targeting these downstream effectors of L1-mediated signaling could provide additional approaches to CRC diagnosis and therapy.
